# Re-production of Bone

**Published:** 1866-02

**Authors:** 


RE-PRODUCTION OF BONE.—M. OLLIER (Paris), who has been for some time experimenting with the periosteum in forming new bone after the old had been removed, has recently removed the upper half of the humerus from a child, six years old, for disease of the shoulder-joint, carefully detaching the periosteum all round, and dividing none of the muscular attachments. The child now, seven months after the operation, is in good health, and the arm is almost perfectly restored.—*Med. and Sur. Rep.*

